# Weight Management for Athletes and Active Individuals: A Brief Review

**DOI:** 10.1007/s40279-015-0401-0

**Published:** 2015-11-09

**Authors:** Melinda M. Manore

**Affiliations:** School of Biological and Population Sciences, Oregon State University, Milam 103, Corvallis, OR USA

## Abstract

Weight management for athletes and active individuals is unique because of their high daily energy expenditure; thus, the emphasis is usually placed on changing the diet side of the energy balance equation. When dieting for weight loss, active individuals also want to preserve lean tissue, which means that energy restriction cannot be too severe or lean tissue is lost. First, this brief review addresses the issues of weight management in athletes and active individuals and factors to consider when determining a weight-loss goal. Second, the concept of dynamic energy balance is reviewed, including two mathematical models developed to improve weight-loss predictions based on changes in diet and exercise. These models are now available on the Internet. Finally, dietary strategies for weight loss/maintenance that can be successfully used with active individuals are given. Emphasis is placed on teaching the benefits of consuming a low-ED diet (e.g., high-fiber, high-water, low-fat foods), which allows for the consumption of a greater volume of food to increase satiety while reducing energy intake. Health professionals and sport dietitians need to understand dynamic energy balance and be prepared with effective and evidence-based dietary approaches to help athletes and active individuals achieve their body-weight goals.

## Key Points

**Table:** 

The strategies include the following: Avoiding severe energy restriction.
Monitoring protein intake, timing, and quality.
Adopting a low-energy dense (ED) diet.
Timing of food intake around exercise and spreading meals/snacks throughout the day.
Monitoring intake of ED beverages.

## Introduction

Weight management is difficult for most individuals, as indicated by the high numbers of overweight and obese individuals in the USA and around the world [1]. Currently, ~66 % of the US adult population is either overweight and/or obese, with ~34 % being obese [2, 3]. Unfortunately, the obesity epidemic is not limited to adults. Currently, 32 % of children and youth between the ages of 2 and 19 years are above the 85 % percentile for body mass index (BMI, kg/m^2^) for age [4], which means that more young athletes will come to their sport fatter than considered desirable for optimal performance.

Although not typically obese or over fat, elite and recreational athletes can also struggle with body-weight and image issues. Depending on the sport, it is not unusual for athletes to want to lose body fat while gaining or maintaining lean tissue. While some athletes appear to be naturally lean, with weight and body size well matched for their sport, others need to change their weight and/or body composition to be competitive. If a child/youth athlete is overweight or obese, they may have a significant amount of weight to lose to be considered competitive and, thus, be pressured by themselves and others to lose weight. Youth sports programs are an optimal time to help young athletes learn to eat for health, performance, and weight management, applying the basic concepts of energy balance. Thus, there is a need for weight-management guidelines to help both elite and recreational athletes of all ages achieve an optimal body weight and composition for their sport and health. This brief review discusses weight issues in athletes, reviews how an athlete might determine a realistic body weight for themselves and their sport, discusses dynamic energy balance in the context of sport, and reviews new methods for predicting weight loss while dieting, and provides dietary strategies an athlete might use to successfully facilitate the maintenance of a healthy body weight or increase fat loss, while maintaining lean tissue. As much as possible, the research presented is from studies using athletes or active individuals. Over-the-counter weight-loss supplements [5] and weight gain in athletes have been covered elsewhere [6].

## Weight Issues in Athletes and Active Individuals

In general, most athletes and active individuals who want to lose weight fall into two categories:

- Those who are overfat or obese based on body-fat levels.
- Those who are already lean, but desire additional body fat loss. Some of these athletes fall into weight-sensitive (e.g., endurance athletes, ski jumping), weight-class (e.g., wrestling, judo), or aesthetically judged (e.g., gymnastics, figure skating) sports [7].

For the athlete with excess body fat, weight loss could improve sport performance and reduce the risk of chronic disease. For example, Borchers et al. [8] found that 21 % of their Division 1 college football players (mean age, 20 years) were obese (≥25 % body fat) and had insulin resistance, while 9 % had metabolic syndrome (all obese). Thus, for these athletes, weight loss could improve performance and prevent the development of serious chronic diseases.

Conversely, many elite and recreational athletes are normal weight or have low body weights, yet they still want to lose weight to improve performance and/or to achieve a body shape for aesthetic reasons. Some of these individuals are young and still growing, which is the least desirable time to severely restrict energy intake while participating in high levels of exercise. In helping these individuals achieve their weight and sport goals, it is imperative that the risk of introducing restrictive and disordered eating behaviors is minimized, especially in those athletes participating in lean build sports [7, 9, 10]. Finally, it can be difficult to manage safe weight loss in athletes who need to meet a designated weight on competition day, such as lightweight rowers, jockeys, or wrestlers. These individuals typically weight cycle, with their weight fluctuating dramatically between the competitive and off seasons [11, 12]. In addition, for athletes in aesthetic sports (e.g., figure skaters, synchronized swimmers, gymnasts), keeping weight low over a competitive season without injury or illness or the use of extreme weight-control methods is also a challenge [13]. Few athletes are naturally light weight enough for these types of competitive sports, so weight loss will be required the weeks or days prior to competition [14].

Regardless of why the athlete needs to lose weight, working with a registered dietitian (RD) trained in sport nutrition can help the athlete identify and reach a realistic goal weight without the use of extreme diets or dangerous weight-loss practices or supplements [7].

## Achieving a Healthy and Competitive Body Weight

Depending on the sport, the weight an athlete can maintain without dieting is typically higher than their competition weight. Thus, many athletes will restrict energy intake to achieve their competitive weight and gain weight in the off season. The ultimate goal is to identify a healthy body weight that the athlete can maintain for most of the year, while minimizing the amount of weight that needs to be lost for competition. This approach reduces the yo-yo dieting that occurs each season. For some sports, trying to maintain a low competition weight throughout the year is not possible or healthy for most athletes. The following questions can help the athlete identify whether the weight they are trying to achieve is realistic and can be maintained without constant dieting [15]. The athlete can determine what weight works best for them during the off season and how much time they need to reach their competitive weight while remaining healthy and injury free.

- Does the goal weight minimize health issues that can increase the risk for injuries [10, 16] and promote good health and eating habits, while allowing for optimal sport training and performance?
- Does the goal weight take into consideration the genetic makeup and family history of body weight and shape?
- Is the goal weight appropriate for age and level of physical development, including normal reproductive function?
- Can the goal weight be maintained without constant dieting or restraining food intake, which could lead to disordered eating or an eating disorder [7, 9]?

Ultimately, the goal is to identify a weight that promotes good health and is ‘reasonable’ to achieve and maintain for most of the year, while keeping the ‘diet for weight loss’ periods short. An athlete who is constantly dieting or repeatedly gaining and losing weight may be trying to achieve an unrealistic body weight, which may place them at risk for disordered eating. A sport dietitian can monitor these athletes to assure they are maintaining healthy eating habits. It also requires that the medical and coaching staff know and can recognize risk factors for disordered eating when they occur and initiate early intervention [9].

## Dynamic Energy Balance

Maintenance of body weight is an indication of being in a state of energy balance where energy intake (total kcal consumed) equals energy expenditure (total kcal expended). However, if the goal is to change energy balance, either for weight gain or weight loss, this static energy balance approach no longer applies since weight is changing. Energy balance is a dynamic process [17], and changing one factor on the energy intake side can also impact the energy expenditure side even without any intentional effort to alter energy expenditure. Thus, numerous factors work together to influence each side of the energy balance equation, which ultimately determines body weight. For example, total energy expenditure will be influenced by total energy intake, dietary macronutrient composition, and the energy density of the diet [17, 18]. These dietary factors can also alter the thermic effect of food [18] and the type of substrates oxidized during exercise [15, 19, 20]. Conversely, exercise type, duration, and intensity can alter total energy intake. For example, high-intensity exercise can blunt appetite-regulating hormones, which may ultimately lead to reduced energy intake [21–23]. Other factors that can confound the assessment of energy balance in an athlete are the total amount of non-sport-related activities (e.g., walking and biking as modes of transportation, yoga, dancing, etc.) [24] and the amount of sitting, standing, and fidgeting an athlete does [25]. While some athletes are very active outside of training for their sport, others become quite sedentary when they are not training, which can decrease energy needs below predicted levels [26]. Finally, assessing energy balance in a highly active athlete can also be challenging. Energy expenditure can be altered due to changes in energy efficiency and body composition with training, which may not be captured unless doubly labeled water (DLW) is used to measure energy expenditure.

In 1958, Wishnofsky [27] concluded that when individuals consumed a low-calorie, high-protein diet for weight loss, 1 pound (lb, 454 g) of weight lost was equal to approximately 3500 kcals. This calculation was based on examining the energy content of a pound of body fat and the research literature at the time examining weight loss in obese individuals participating in controlled research studies. Many health professionals make a common mistake when explaining energy balance to athletes and active individuals. They assume that changing either side of the equation by 3500 kcal (7700 kJ) will always result in a pound of weight gained or lost, without considering all the other factors that might change as energy intake or energy expenditure is altered. Swinburn and Ravussin [28] gave a classic example to illustrate the fallacy of this concept. Using a 75-kg man, they demonstrated how weight would change if this individual consumed an extra 100 kcal/day (~420 kJ) for 40 years [28]. The static energy balance equation would calculate the amount of extra energy consumed to equal ~1.5 million kcal with an estimated weight gain of 417 lbs (~190 kg) over the 40-year period. Yet, intuitively, health professionals know this would not happen. The static energy balance calculation does not take into account the increase in energy expenditure that would occur as weight is gained. As weight increases, resting metabolic rate (RMR) and energy expenditure would also increase, since there is a greater energy cost in maintaining and moving a larger body. What would actually happen is that after a short period of positive energy balance, body weight would increase, resulting in an increase in energy expenditure that will eventually balance the increased energy intake. Thus, the individual would eventually achieve energy balance and become weight stable at a higher body weight, which might be a realistic weight gain of ~6 lbs (~2.7 kg). However, to maintain this larger body size, the individual would need to continue to eat these additional kcals each day. Of course, the amount of actual weight gained will depend on a number of individual factors, extra kcal consumed, composition of the diet [29], body composition, type of exercise in which the individual is engaged, and overall energy expenditure.

**Fig. 1:**
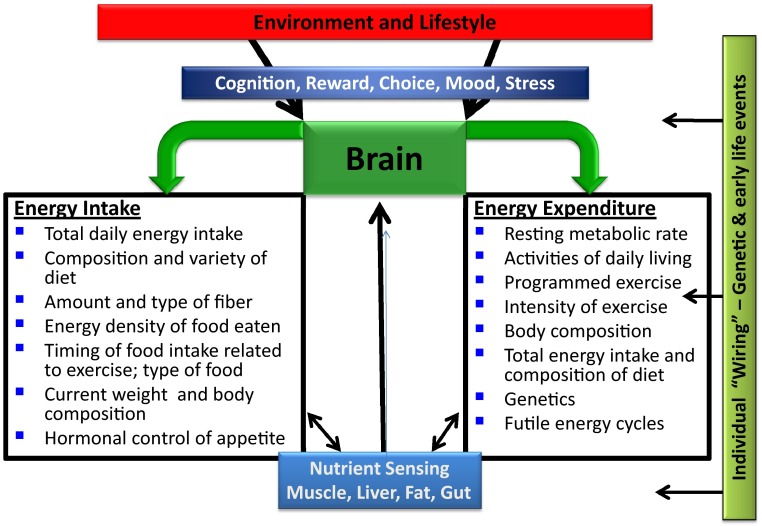
Factors regulating/influencing energy balance

The concept of dynamic energy balance and some of the key factors that influence each side of the energy balance equation is illustrated in Fig. 1. How an individual responds to changes in each factor will depend on genetics, changes in regulatory hormones that control energy balance and appetite, gut health, and the food and exercise environment that can drive eating, exercise, and body composition. For further details, please see Galgani and Ravussin [17].

## Predicting Weight Loss with Dieting

Wishnofsky’s static energy balance equation is still widely used in the research literature and given to athletes as a guide for weight loss [7, 30]. However, we now know that weight loss changes over time with the same level of negative energy imbalance [31]. The energy deficit required for weight loss is less at the beginning of a diet (e.g., <3500 kcal/lb or 7700 kcal/kg) [32], which is attributed to changes in glycogen, protein, and fluid balance [33]. This point was demonstrated by Heymsfield et al. [32], as they found that when overweight (BMI ~28 kg/m^2^) sedentary men and women restricted energy intake by 25 %, the energy deficit required for weight loss was ~2200 kcal/lb during the first 4 weeks of the diet. As the time on the diet increased, the energy deficit required for weight loss also increased and approached Wishnofsky’s ‘rule’ of 3500 kcal/lb by 6 months [32]. Thus, the degree and rate at which weight loss occurs on a diet will depend on numerous factors, including the level of energy restriction, composition of the diet [34, 35], total daily energy expenditure, and initial body composition [36–40].

To better predict weight change in response to changes in energy intake or expenditure, one must account for the dynamic energy imbalances that occur. To address this issue, researchers have developed mathematical models to simulate how alterations in energy deficit result in adaptations of fuel selection and energy expenditure to better predict body weight and composition changes [33]. One mathematical model has been developed by Hall et al. [41] at the National Institutes of Health (NIH) (http://bwsimulator.niddk.nih.gov) and a second model has been developed by Thomas et al. [42] at the Pennington Biomedical Research Center (PBRC) (http://www.pbrc.edu/research-and-faculty/calculators/weight-loss-predictor/). As one changes energy intake or expenditure, these models take into account changes in RMR, fat and lean tissue mass, voluntary physical activity, spontaneous physical activity, the thermic effect of food, and the energy costs of fat and protein synthesis. Using the Thomas prediction model for weight change and data from the CALORIE I study conducted in overweight individuals [32, 36], these investigators showed that their model predicted within 2.2 kg of the actual weight loss, while the Wishnofsky approach had an 11-kg bias [31]. However, it is important to understand that these prediction models were developed using the results from weight-loss studies with overweight and obese individuals. Thus, the application to athletes and active individuals who are leaner and capable of much higher energy expenditures needs to be considered. Regardless, these models can help the health professional do a better job of estimating the time required for weight changes to occur and to develop more realistic weight-loss goals for a given time period. Table 1 demonstrates the difference in time needed for a designated weight loss change for an overweight rower using the Hall et al. [41] model compared with the Wishnofsky rule.

**Table 1:** Case study of a collegiate male crew athlete desiring fat loss: time for weight-loss change using two different approaches predict weight loss^a^

Approach	Time needed to achieve desired weight loss
NIH [41] body weight simulator: http://bwsimulator.niddk.nih.gov. This model allows input of demographic data (initial weight, sex, age, height, measured RMR, % of energy from CHO, initial sodium, % body fat, and PAL. If measured values are not available, RMR, % body fat, and PAL are estimated for the individual	Lifestyle changes made: No change in exercise training program; 500 kcal/day reduction in energy intake from baseline Outcome: Simulator estimates energy intake at 4229 kcal/day based on a PAL of 1.9 and an initial RMR of 2226 kcal/day. The NIH weight simulator estimates it would take 95–105 days to reach his goal weight, with body fat reducing to 14.4 % and BMI to 27.5 kg/m^2^. Because diet composition can change outcomes, the simulator allows the user to determine the percent of energy from CHO desired. In this simulation, 50 % of energy from CHO was used. Sodium levels can also be adjusted
Wishnofsky’s rule [27]: Assume that a caloric reduction in 3500 kcal will result in 1 lb (454 g) of weight loss	Lifestyle changes made: No change in exercise training program; 500 kcal/day reduction in energy intake from baseline Outcome: The health professional needs to estimate energy needs. If the Harris–Benedict equation is used to estimate RMR (2422 kcal/day) and a PAL of 1.9 is applied (same as above), the energy needs are estimated at 4602 kcal/day. The health professional would need to estimate activity level. With a 500 kcal/day reduction, 1 lb/week is the estimated weight loss. Using these assumptions, it would take 12 weeks (84 days) to lose 12 lbs (5.45 kg)
Other comments: Composition of diet matters when energy is restricted in an athlete. Adequate protein and CHO can help preserve lean tissue and replace glycogen. How diet composition changes can also impact energy expenditure (e.g., thermic effect of food). If training level changes weekly, the energy intake needs to reflect these changes. During periods of competition, the degree of energy restriction may need to be altered to maintain energy levels and prevent fatigue	

*BMI* body mass index, *CHO* carbohydrate, *NIH* National Institutes of Health, *PAL* physical activity level

^a^Male crew member (20 years, 242 lbs [110 kg], 77 inches [196 cm], 16.5 % body fat, body mass index 28.7 kg/m^2^) wants to lose 12 pounds (5.45 kg) in 40 days. The goal weight is 230 lbs (104.5 kg). He currently trains 6 days per week for 1–2 h/day, which consists of rowing on the water or indoors and strength training (2 days per week)

## Dietary Strategies for Weight Loss or Maintenance

What changes in diet and exercise behaviors will produce the desired body-weight and composition changes while being sustainable and manageable by the active individual? The following section highlights evidence-based diet and lifestyle recommendations for athletes and active individuals who are interested in losing weight (e.g., fat mass), maintaining lean tissue, and/or preventing weight regain. This section does not specifically address changes in exercise strategies or training routines, since the coach typically determines these for the athlete. For athletes who are already very active, they will need to rely more heavily on the dietary and lifestyle strategies listed below to achieve weight loss. For athletes who are less active or not in training, increasing physical activity in addition to dietary manipulations may be necessary.

### Avoid Severe Energy Restriction

It is tempting to severely restrict energy intake to get quick weight-loss results. However, this approach, combined with an intense endurance and strength-training program, can actually increase metabolic adaptations that slow weight loss and diminish the additive effects of these two factors on weight loss [37]. Thus, this approach should be avoided [7, 10, 13, 36]. It is important to remember that with negative energy balance, lean, fit individuals can quickly lose lean tissue if energy is restricted too dramatically [39]. For example, Pasiakos et al. [43] placed active military personnel (BMI 25 ± 1 kg/m^2^) on a 40 % energy-restricted diet for 30 days, while being fed the recommended dietary allowance (RDA) for protein (0.8 g/kg/body weight). Of the 3.3 kg lost during this time (4.2 % body weight), 58 % was lean tissue (1.9 kg). In contrast, when Redman et al. [36] placed sedentary overweight individuals (BMI 27.8 kg/m^2^) on a 25 % energy-restriction diet for 3 months, they lost 6 kg, with only 33 % coming from lean tissue (2 kg). In addition, Garthe et al. [44] showed that slower, more reasonable weight loss in athletes (~0.7 % loss of body weight/week) helped preserve lean tissue while improving strength gains compared with more severe weight loss (1.4 % weight loss/week). Finally, severe energy restriction during times of high exercise has a number of other negative performance and health consequences for the athletes, as follows [10, 15]:

- Decreased sport performance effects due to decreased muscle strength, glycogen stores, concentration, coordination and training responses, and increased irritability.
- Increased negative health consequences, such as injury due to fatigue, loss of lean tissue, and poor nutrient intakes, including essential nutrients, due to limited food intake.
- Increased risk of disordered eating behaviors due to severe energy restriction.
- Increased risk of dehydration, especially if the diet is ketogenic.
- Increased emotional distress due to hunger, fatigue, and stress related to following an energy-restricted diet.

Thus, for the athlete and active individual who already have a training or fitness program, it is better to moderately restrict energy intake (e.g., 500–700 kcal/day) and take longer to reach the weight loss goal [37, 44]. This approach also allows the time required to adapt to new dietary habits while making sure adequate energy is available for exercise training.

### Monitor Protein Intake, Quality, and Timing

When energy is restricted, it is easy for protein intake to decrease at the same time that protein needs to increase with energy restriction to help preserve skeletal muscle integrity, especially in physically active individuals [45]. In general, the protein needs of athletes are higher (1.4–1.7 g/protein/kg) [46] than that recommended by the RDA (0.8 g/protein/kg) for non-active individuals [47]. The amount of additional protein needed will depend on the volume and type of exercise and the level of energy restriction [45]. For example, Mettler et al. [34] demonstrated that increasing dietary protein during periods of severe energy restriction can help maintain lean tissue in active individuals participating in strength training while dieting. For 1 week, they placed 20 healthy resistance-trained male athletes (body fat 16–17 %, BMI 23–24 kg/m^2^) on an energy-restricted diet (60 % of habitual energy intake). During this time, they were randomly assigned to either a control (1 g/protein/kg; *n* = 10) or treatment group (2.3 g/protein/kg; *n* = 10) [34]. Results showed that loss of lean mass was greater in the control group (–1.6 kg in 1 week) than in the treatment group (–0.3 kg). Thus, the higher protein intake (~35 % of energy intake) helped preserve lean tissue when energy intake was severely restricted for a short time.

In addition to consuming more protein overall, athletes need to consume adequate high-quality protein throughout the day, but especially after exercise and at breakfast [29]. Spreading protein intake throughout the day can benefit the athlete trying to lose weight by ensuring that adequate protein is constantly available for building, repair, and maintenance of lean tissue. Second, higher protein diets have been associated with increased satiety and reductions in energy intake. For example, Weigle et al. [48] reported a decrease in energy intake (–441 ± 64 kcal/day) over a 12-week period in healthy sedentary individuals (BMI 26.2 ± 2.1 kg/m^2^) fed an ad libitum high-protein diet (30 % energy from protein, 20 % fat, and 50 % carbohydrate) compared with an isocaloric lower protein diet (15 % of energy from protein). Although most athletes consume plenty of protein [15], they may not be strategic about getting this protein after exercise and spreading it out across the day. It may be more typical for the majority of the energy and protein to be consumed in a large meal at the end of the day.

### Adopt a Low-Energy Dense Diet Plan

A low-ED diet is high in whole fruits, vegetables, and grains, and incorporates low-fat dairy, legumes/beans, and lean meats. Overall, the diet is lower in fat and reduces or eliminates ED beverages, especially sweetened beverages and alcohol. This high-fiber, high-water, low-fat diet means an individual can consume a greater volume of food for an overall lower energy intake and still feel satiated. The energy density of a diet or a food is determined by measuring the amount of energy (kcal) for a given amount (g) of food (kcal/day). Evidence shows that a low-ED eating plan is effective at reducing energy intake, facilitating weight loss, and preventing weight regain, and maintaining satiety in well-controlled feeding studies and in free-living conditions [49, 50] For example, Bell et al. [51] examined the effectiveness of a low-ED eating plan on total energy intake and weight loss. They found that when they fed three different levels of ED diets, the women ate a similar amount and weight of food; however, on the lowest low-ED diet condition, participants consumed 30 % less energy than the high-ED diet. Furthermore, the women did not report any differences in hunger and fullness ratings or enjoyment of the meals across test conditions. In a follow-up study, Rolls et al. [52] examined the effect of changing portion size, energy density or a combination of the two conditions on total energy intake over 2 days. Energy density was altered by changing the portions of vegetables in entrées and by substituting low-fat foods/ingredients for full-fat foods (e.g., skim milk for whole milk). They found that energy density and portion size independently altered energy intake. When portion size was reduced by 25 %, energy intake decreased by −231 kcal/day (10 % decrease); however, reducing energy density by 25 % decreased energy intake by −575 kcal/day (24 % decrease). When both energy density and portion sizes were reduced simultaneously, energy intake decreased by 32 %. Thus, reducing portion sizes and energy density reduces energy intake; however, just reducing the energy density of the foods consumed reduces energy intake more than reducing portion sizes [52]. Subsequent research has shown this weight loss approach also works in longer dietary interventions. Ello-Martin et al. [53] showed that obese women counseled to consume a low-ED diet (*n* = 35) for 1 year lost 20 % more weight (–7.9 kg) than those counseled to reduce fat intake only (*n* = 36; –6.4 kg). Dietary fat intake was similarly reduced in both groups, but those in the low-ED group reported significantly lower ratings of hunger. Physical activity did not differ between the groups, with mean step counts at 8735 per day.

Currently, no published research has reported using a low-ED diet for weight loss in athletes; however, researchers have observed that female athletes with exercise-associated menstrual dysfunction who consume low-ED diets have inadequate energy intake to match energy expenditure [54, 55]. This research suggests that for highly active females, a low-ED diet does not provide enough energy to cover the cost of exercise and reproductive function. The satiating effect of these diets combined with the hunger-blunting effects of intense exercise may contribute to the under-eating of these athletes.

Overall, reducing the ED of the diet is more effective at lowering energy intake than is reducing portion size, without affecting hunger, fullness, or enjoyment of the food. For athletes trying to lose weight, this has important implications. It may be easier for an active individual to consume a similar amount of food and focus on changing the energy density rather than the portion sizes. This approach reduces hunger and increases adherence to the weight-loss diet plan. Finally, following a lower-ED diet could help the athlete maintain their weight loss. In summary, a key component of a low-ED eating plan is to increase the intake of foods high in water and fiber that promote satiation, while reducing both high-fat foods (i.e., potato chips, cheese, cookies) and low water and fiber foods (i.e., baked tortilla chips, pretzels). The low-ED eating plan also increases total fiber intake, which helps individuals achieve the recommended intakes.

### Timing of Food Intake Around Exercise and Spreading Meals/Snacks During the Day

For the athlete, timing of food intake around exercise training and spreading food intake throughout the day will ensure that the body has the energy and nutrients needed for exercise and the building and repair of lean tissue. This approach can also prevent the athlete from becoming too hungry and consuming foods or beverages not on their diet plan. Unfortunately, when athletes attempt to lose weight, they often use unhealthy weight-loss practices such as fasting or skipping meals, severe energy restriction, and dehydration [13, 14]. When athletes are concerned about weight, especially female athletes, they restrict meals, especially breakfast. For example, Erdman et al. [56] reported that nearly all (98 %) of their elite-level Canadian athletes (mean age 20.6 years, 36 % male) consumed breakfast, while Shriver et al. [57] found that only 23 % of their Division I college-level female athletes consumed breakfast. The majority of the athletes in the Shriver et al [57] study reported their diets to be fair/poor, ate the majority of their calories at dinner, and expressed difficulties in maintaining weight. In addition, one-third of the athletes wanted to lose weight. Thus, breakfast skipping may be a result of not making breakfast a priority or hoping that skipping a meal will help reduce overall caloric intake.

For the athlete, the breakfast or mid-morning meal is especially important because it can provide needed carbohydrates to help replenish glycogen after an overnight fast and provide fuel for exercise. For those athletes who participate in early-morning workouts, eating a light snack prior to practice and a nutritious breakfast after practice will assure that adequate nutrients are consumed, especially carbohydrate and protein. For example, Carlsohn et al. [58] found that, for junior elite triathletes, breakfast provided 21 and 28 % of the daily carbohydrate intake during moderate- and high-intensity training weeks, respectively. Thus, skipping breakfast would mean that either total daily carbohydrate intake would be lower, potentially impacting exercise performance, or that other meals and snacks would have to provide this carbohydrate intake. Fortunately, it is easy to consume a low-ED, high-nutrient dense breakfast by including low-fat, high-quality protein (e.g., low-fat dairy or soy products, egg whites, lean meats) and high-fiber, carbohydrate-rich foods (e.g., whole grains, fruits).

Finally, refueling after exercise is still important for the athlete during weight loss. Thus, the post-exercise dietary routine needs to include fluids for rehydration, carbohydrates in the form of low-ED foods (e.g., whole fruits and vegetables, whole grains, legumes/beans) to replenish glycogen, and high-quality low-fat protein for building and repair of lean tissue. Because many athletes may not have these foods readily available after exercise, they must plan ahead and strategically use sport foods and/or health snacks to meet their energy and nutrients needs while staying within their diet plan. A sport dietitian can teach the athlete how to shop for, select, and prepare low-ED foods. Remember, the use of low-ED foods for refueling is best during training periods when there is adequate time between exercise sessions to replace muscle glycogen. During periods of competition, higher-ED foods may be required if glycogen replacement needs to occur in less than 24 h.

### Reduced Consumption of Energy Dense Beverages

Consumption of ED beverages and alcohol add energy to the diet, but show reduced satiety and incomplete energy compensation [59]. For some athletes, the elimination of ED beverages from their diet may help them achieve their weight loss goals without making any other dietary changes. For these athletes, sweetened beverages (e.g., sport drinks) should be limited to what is needed for hydration and fueling when participating in exercise and sport.

## Conclusions

For the athlete and active individual, management of weight can be difficult when good-tasting food is so convenient, abundant, and relatively inexpensive. Although athletes expend high amounts of energy in exercise, they may still need to monitor diet and lifestyle to maintain a competitive body weight. If an athlete needs to lose weight, working with a supportive team (e.g., coach, sports medicine team, and sport dietitian) will help ensure success. In addition, the sport dietitian can help make daily meal plans, address nutrition and sport supplements and health issues, and make sure the athletes is fueled for their sport. To provide a consistent message to the athlete, all health professionals need to understand the many physiological and environmental factors influencing body weight and energy balance. They also need to provide the same key messages to athletes that are outlined in Table 2. This will improve their ability to design individualized and realistic weight-management programs.

**Table 2:** Dietary strategies for weight loss or maintenance in athletes and active individuals

Weight loss/management strategies	‘Bottom line’
Use a dynamic energy balance approach to predict weight loss based on changes made in diet and exercise	Two mathematical models [41, 42] have been developed to help predict weight gain/loss based on changes in lifestyle using the dynamic energy balance approach: 1. NIH model: http://bwsimulator.niddk.nih.gov 2. Pennington model: https://www.pbrc.edu/research-and-faculty/calculators/
Avoid severe energy restriction	When energy restriction is too severe, lean, fit individuals quickly lose lean tissue. Severe energy restriction can also compromise health and performance due to decreased muscle strength, glycogen stores, concentration, and training response. Risk of injury can increase due to fatigue and loss of lean tissue [10]. Energy intakes below 1500 kcal/day are typically below the RMR of most athletes and should be avoided. Even a small female (50 kg [110 lbs]; 152 cm [60 inches]) has an RMR of ~1300 kcal/day or higher
Maintain a higher protein intake when energy is restricted	When energy intake is reduced, protein intake can also be reduced. During periods of weight stability, active individuals are recommended to consume from 1.4 to 1.7 g/protein/kg/day [46]. Although the exact amount of protein required during energy restriction has not been established and would depend on level of energy restriction and type of activity program, 25–35 % of energy from protein should be adequate to attenuate losses in skeletal muscle [60]. This typically translates to a diet containing >1.6 g protein/kg/day
Follow a low-ED diet to increase satiety when energy is restricted	Following a low-ED diet plan can increase satiety while lowering total energy intake [52]. A low-ED diet is high in whole fruits and vegetables, whole grains, and incorporates low-fat dairy, legumes/beans, and lean meats
Time food intake around exercise and throughout the day	Timing of food intake around exercise training and spreading food intake throughout the day will assure the body has the energy and nutrients needed for exercise and the building and repair of lean tissue
Monitor consumption of ED beverages	Consumption of energy dense beverages and alcohol add energy to the diet, but show low satiety and incomplete energy compensation [59]. Use of energy-containing sport beverages during exercise in an attempt to help maintain blood glucose and hydration levels is still recommended

*ED* energy dense, *NIH* National Institutes of Health, *RMR* resting metabolic rate
